# Challenging Perioperative Management of a MEN2A Syndrome Patient Complicated by Eisenmenger Syndrome

**DOI:** 10.4274/TJAR.2025.241768

**Published:** 2025-03-21

**Authors:** Amit Rastogi, Gaurav Agarwal, Sumit Sachan, Aditya Kapoor, Preeti Dabadghao

**Affiliations:** 1 Sanjay Gandhi Postgraduate Institute of Medical Sciences, Department of Anaesthesiology, Uttar Pradesh, India; 2 Sanjay Gandhi Postgraduate Institute of Medical Sciences, Department of Endocrine Surgery, Uttar Pradesh, India; 3 Sanjay Gandhi Postgraduate Institute of Medical Sciences, Department of Cardiology, Uttar Pradesh, India; 4 Sanjay Gandhi Postgraduate Institute of Medical Sciences, Department of Endocrinology, Uttar Pradesh, India

**Keywords:** Cardiovascular and thoracic anaesthesia, Eisenmmenger syndrome, intensive care, MEN2A, perioperative care, pheochromacytoma

## Abstract

Multiple endocrine neoplasia type 2A (MEN2A), is associated with pheochromocytoma and medullary carcinoma of the thyroid. A surgical procedure in these patients can be complicated if they have any congenital heart disease (CHD). Nowadays, CHD patients are increasingly presenting at advanced age for non-cardiac surgeries, posing unique challenges to anesthesiologists. We hereby present a 44-year-old male with Eisenmenger syndrome (ES) and MEN2A, scheduled for bilateral adrenal excision and thyroidectomy. Patients with ES require meticulous and goal-directed management during non-cardiac surgery, depending upon pulmonary hypertension, cyanosis, and right ventricular dysfunction.

**Main Points**

• Optimal perioperative medical management is essential for hemodynamics and shunt management in Eisenmenger syndrome (ES) patients for non-cardiac surgery.

• Anemia correction in the postoperative period is essential in patients with ES as they require a relatively higher hemoglobin concentration than healthy adults to compensate for chronic hypoxemia.

• Point-of-care ultrasound, intermittent non-invasive ventilation, and awake proning are crucial in managing refractory hypoxemia in Eisenmenger patients for non-cardiac surgery.

• We aimed to share our perioperative experience of adult ES patients posted for two major non-cardiac surgeries in one sitting, together.

## Introduction

Congenital heart disease (CHD) patients may develop Eisenmenger syndrome (ES) as age advances, which leads to an increase in perioperative complications when non-cardiac surgery is performed. The risks of surgery in patients with ES vary according to the nature, complexity, and urgency of the procedure (emergency cardiac surgery versus routine abdominal surgery versus dental procedures).^1^ Ammash et al.^2^ described 24 patients with ES who underwent 28 non-cardiac surgeries. They found that the perioperative mortality rate was 7%. Bennet et al.^1^ examined a cohort of 33 patients with ES and reported systemic hypotension in 26%, oxygen desaturation in 17%, and 30-day mortality of 3.8%. We hereby present a clinical case of a patient with ES undergoing two major non-cardiac surgeries. The complete procedure was explained to the patient, and informed consent was obtained.

## Case Report

**Figure 1:**
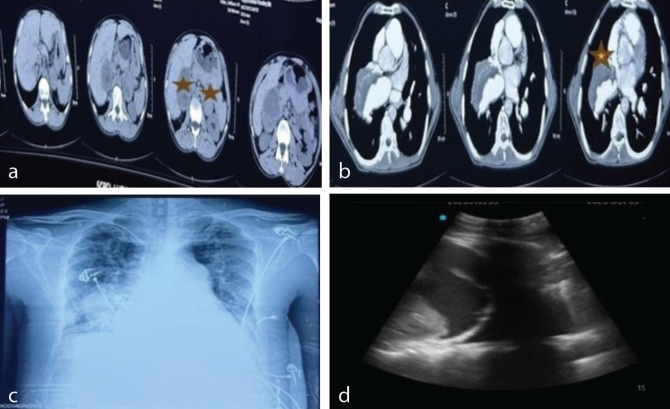
(a) Preoperative CT scan of the abdomen showing bilateral adrenal tumors (see star). (b) Preoperative CT Pulmonary Angio showing thrombus in the right pulmonary artery (see star). (c) Postoperative chest skiagram showing basal atelectasis and blunting of costophrenic angles. (d) POCUS lung showing pleural effusion. CT, computed tomography; POCUS, point-of-care ultrasound.

A 44-year-old male with a body weight of 45 kg was posted for bilateral adrenal mass excision and total thyroidectomy. The patient had been diagnosed with CHD and pulmonary hypertension for 11 years. The echocardiogram revealed a large peri membranous ventricular septal defect (16 mm), with bidirectional shunting, dilated main pulmonary artery, and right pulmonary artery (RPA) thrombus with pulmonary artery hypertension (right ventricular systolic pressure of 136 mmHg). The high-resolution computed tomography (CT)-thorax of the patient showed a heterogeneous right pulmonary hilar mass and bilateral adrenal lesion (Figure 1a). The CT angio revealed a thrombus (large, eccentric) Figure 1b.

The patient was informed of his comorbid condition and associated high risk. The patient’s metabolic equivalents (METs) were six, and the patient was kept on tab rivaroxaban 20 mg, tab tadalafil 20 mg tab ambrisentan 5 mg OD, tab prazosin 2.5 mg OD, and tab furosemide/amiloride (5/40). The anaesthetic plan included an epidural catheter at the T10-11 region and 0.5 mg of morphine in the epidural space. The patient underwent general anaesthesia with muscle relaxation. The patient had hemodynamic fluctuations during surgical manipulations of adrenal tumours, and they were managed by titration of infusion of injection (inj.) nitro-glycerine [5-20 µg minimum (min)] initially followed by inj. nitroprusside (0.5-10 µg kg^-1^ min^-1^), in the final stages of tumour excision. Following adrenalectomy of bilateral adrenal tumours, the patient had hypotension and a fall in oxygen saturation down to 80%. Noradrenaline infusion (0.01-0.3 µg kg^-1^ min^-1^) was titrated to maintain hemodynamics, and an intravenous (IV) inj. of sildenafil 10 mg was given for oxygenation. Inj. hydrocortisone (200 mg 24 h) infusion was initiated after persistent hypotension following bilateral adrenalectomy, suspecting acute adrenocortical insufficiency as a cause and the fluid status was optimized. During thyroidectomy, the patient again had a fall in saturation. IV inj. sildenafil 10 mg was administered slowly, and saturation improved to 88-90%. After thyroidectomy surgery, the patient was transferred to the post-anaesthesia care unit.

The patient was extubated the day after surgery. Initially, the patient-maintained oxygen saturation (SpO_2_) of around 88-90% on a face mask with slightly higher pCO_2_, and normal lactates. Gradually, the patient showed progressive hypoxemia, and Intermittent non-invasive ventilation (NIV) was started. On POD-3, hypoxemia persisted with SpO_2_ of 68-70% along with tachypnoea. The patient’s hemoglobin was 7.5 g dL^-1^, and a packed red blood cell (PRBC) transfusion was planned. The patient was kept on: tab tadalafil 20 mg, tab ambrisentan 5 mg OD, inj. unfractionated heparin (UFH) IV 5000 units TDS, and tab furosemide/amiloride (5/40).

**Figure 2:**
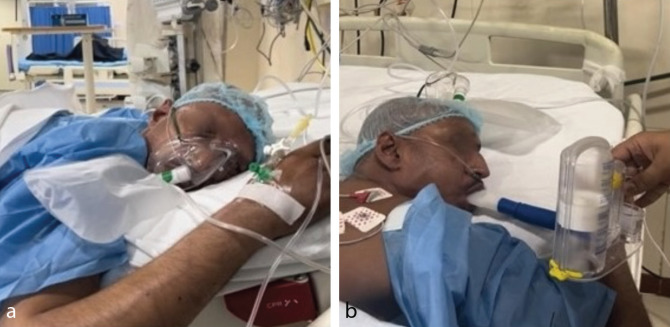
(a) Patient undergoing prone ventilation. (b) Patient undergoing incentive spirometry while in prone position.

A total of 4 PRBCs were transfused in 4 days. However, the patient’s hypoxemia persisted, and intermittent NIV was continued. The NT pro-BNP levels were >10,000 pg mL^-1^. Chest X-ray revealed basal atelectasis and blunting of costophrenic angles, Figure 1c. Point-of-care ultrasound (POCUS) revealed moderate pleural effusion on the right side with moderate ascites: Figure 1d. Inj. furosemide 40 mg BD was started for negative fluid balance, UFH was replaced with tab rivaroxaban 20 mg, and inj. albumin infusion of 40 g in 24 hours was initiated. For persistent hypoxemia, prone positioning and incentive spirometry were initiated (Figure 2a, 2b). The patient SpO_2_ (80-85%) improved with the prone positioning. The patient’s tachypnoea gradually settled and SpO_2_ on nasal prongs returned to 88-90% at POD-15. POCUS revealed minimal right-sided pleural effusion. There were decreasing trends of NT pro-BNP levels (10,000 pg mL^-1^, 5,500 pg mL^-1^, 2,200 pg mL^-1^, and 1,305 pg mL^-1^). Gradually, the patient was weaned off oxygen support and transferred to the ward on room air.

## Discussion

Mortality with cyanotic heart disease or pulmonary arterial hypertension following non-cardiac surgery is around 7% to 10%.^2, 3^ In patients with ES, multiple organs are affected due to chronic cyanosis, collaterals, platelet dysfunction, and alterations in coagulation.^4^ When METs are less than 4, it equates to an inability to climb two flights of stairs and is associated with a greater incidence of postoperative cardiac events.^5^ The average peak VO_2 _of a patient with ES is 11.5 mL kg^-1^ min^-1^, which equates to less than 4 METs and indicates a higher risk.^6^ Our patient has >6 METs; however, MET information is challenging to interpret in PAH-CHD patients who underplay their symptoms.

Maintenance of oxygenation with increased systemic vascular resistance via IV noradrenaline infusion was essential. Pulmonary vascular resistance is usually fixed in patients with shunt reversal; however, IV phosphodiesterase-5 (PDE-5) inhibitors have given us leverage, during the surgery to maintain acceptable saturation. PDE-5 inhibitors like tadalafil in patients with ES are well tolerated and significantly improve exercise capacity, functional class, systemic oxygen saturation, and pulmonary hemodynamics.^7^ The endothelin receptor antagonist, ambrisentan, showed promising results in patients with Eisenmenger’s in terms of 6-minute walking distance and a reduction in the pulmonary vascular resistance index and mean pulmonary artery pressure.^8^

Patients with ES have structural and functional changes in the pulmonary vessels attributed to the thrombotic phenomenon. These characteristic structural changes of pulmonary hypertension occur in the neo-muscularized small arteries and larger vessels, whereby they may dilate and become aneurysmal.^9^

The universal transfusion trigger is 8 g dL^-1^; however, cyanotic patients have relative anemia. A total of 4 PRBCs were transfused to achieve hemoglobin of 12 g dL^-1^, close to the pre-operative level of 14 g dL^-1^. Patients with ES require a higher hemoglobin concentration than healthy adults to compensate for the chronic hypoxemia (secondary erythrocytosis).^6^ However, when we searched the literature regarding transfusion trigger blood in ES, we found a paucity of descriptions of transfusion triggers in ES. The POCUS, indicated the pleural effusion and ascites, which are indicative of right-sided heart failure, so aggressive IV diuretic therapy, along with inj. albumin infusion (40 g in 24 hours) and inj. milrinone infusion (0.375-0.75 µg kg^-1^ min^-1^) were initiated. NT pro-BNP levels are a sensitive indicator of RV dysfunction. Our diuretic therapy, PDE-5 inhibitors, and endothelin receptor antagonist therapy showed reduced trends in NT pro-BNP levels.^10^ During the entire perioperative course, lactate and carbon dioxide pressure was within the normal range, which could be attributed to our vigilant monitoring and guided clinical actions. Our patient’s refractory hypoxemia was the biggest hurdle to recovery. In such circumstances, ventilation in the prone position induces alveolar recruitment and reduces the right ventricle afterload.^11^

## Conclusion

The adult ES patient can be successfully managed with meticulous planning, optimal perioperative management of shunt function, correction of relative anemia, and planned ventilatory strategies.

## Ethics

**Informed Consent:** The complete procedure was explained to the patient, and informed consent was obtained.
